# Antitubercular potential and pH‐driven mode of action of salicylic acid derivatives

**DOI:** 10.1002/2211-5463.13944

**Published:** 2024-12-03

**Authors:** Janïs Laudouze, Thomas Francis, Emma Forest, Frédérique Mies, Jean‐Michel Bolla, Céline Crauste, Stéphane Canaan, Vadim Shlyonsky, Pierre Santucci, Jean‐François Cavalier

**Affiliations:** ^1^ CNRS, LISM, IMM FR3479 Aix Marseille Univ France; ^2^ INSERM, SSA, MCT Aix Marseille Univ France; ^3^ Laboratory of Cancer Epigenetics, Faculty of Medicine, ULB‐Cancer Research Center (U‐CRC), Institut Jules Bordet Université libre de Bruxelles (ULB) Belgium; ^4^ IBMM, CNRS, ENSCM Univ Montpellier France; ^5^ Laboratory of Physiology and Pharmacology, Faculty of Medicine Université libre de Bruxelles Belgium

**Keywords:** acidic environments, antibiotics, intrabacterial pH homeostasis, iodinated phenolic acids, *Mycobacterium tuberculosis*, weak acids

## Abstract

In the search for new antituberculosis drugs with novel mechanisms of action, we evaluated the antimycobacterial activity of a panel of eight phenolic acids against four pathogenic mycobacterial model species, including *Mycobacterium tuberculosis*. We demonstrated that salicylic acid (SA), as well as the iodinated derivatives 5‐iodo‐salicylic acid (5ISA) and 3,5‐diiodo‐salicylic acid (3,5diISA), displayed promising antitubercular activities. Remarkably, using a genetically encoded mycobacterial intrabacterial pH reporter, we describe for the first time that SA, 5ISA, 3,5diISA, and the anti‐inflammatory drug aspirin (ASP) act by disrupting the intrabacterial pH homeostasis of *M. tuberculosis* in a dose‐dependent manner under *in vitro* conditions mimicking the endolysosomal pH of macrophages. In contrast, the structurally related second‐line anti‐TB drug 4‐aminosalicylic acid (PAS) had no pH‐dependent activity and was strongly antagonized by l‐methionine supplementation, thereby suggesting distinct modes of action. Finally, we propose that SA, ASP, and its two iodinated derivatives could restrict *M. tuberculosis* growth in a pH‐dependent manner by acidifying the cytosol of the bacilli, therefore making such compounds very attractive for further development of antibacterial agents.

## Graphical abstract

Salicylic acid (SA), its two iodinated analogues 5ISA and 3,5diISA, as well as the anti‐inflammatory drug aspirin (ASP), are anti‐TB drugs that disrupt the intrabacterial pH homeostasis of *Mycobacterium tuberculosis* under *in vitro* conditions mimicking the endolysosomal pH of macrophages.

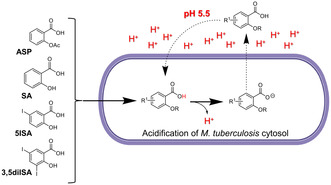

**Abbreviations**
2M,5IBA: 5‐iodo‐2‐methoxy benzoic acid
2IFA: 2‐iodo‐ferulic acid
3,5diISA: 3,5‐diiodo‐salicylic acid
5ISA: 5‐iodo‐salicylic acid
ASP: aspirin
BA: benzoic acid
CC_50_
: cytotoxic concentrations leading to 50% macrophages cell death
GA: gallic acid
IBpH: intrabacterial pH
IGA: 2‐iodo‐gallic acid
MIC_90_
: minimum inhibitory concentration leading to 90% bacterial growth inhibition
PA: phenolic acids
PAS: 4‐amino‐salicylic acid/*para*‐aminosalicylic acid
SA: salicylic acid
TB: tuberculosis
*t*FA: *trans*‐ferulic acid

Tuberculosis (TB), caused by its aetiologic agent *Mycobacterium tuberculosis*, remains a global public health issue worldwide. The latest World Health Organization report estimates that in 2023, 1.25 million people died from TB and approximately 10.8 million people contracted the disease [1].

The current standard drug regimen, composed of isoniazid (INH), rifampin (RIF), ethambutol (EMB), and pyrazinamide (PZA), is generally effective with an estimated average success rate of 88% on TB‐susceptible cases [1]. However, with four distinct drugs to be taken for at least 6 months, this lengthy and toxic regimen often affects compliance, leading to treatment failures and the emergence of drug‐resistant strains [1]. Indeed, recent observations by the World Health Organization suggest that the number of drug‐resistant cases (e.g., RR‐TB; MDR‐TB; and XDR‐TB) is increasing in many areas of the world, leaving clinicians with very few successful therapeutic interventions [1]. In that context, the discovery and/or the development of chemical entities that can kill *M. tuberculosis* more efficiently is urgently needed to better control the global TB pandemic [2].

Phenolic acids (PA), also known as phenol carboxylic acids, are a specific class of aromatic compounds that contain both a phenolic ring and a carboxyl functional group. This class of compounds represents the simplest polyphenols in terms of chemical structure, and some PA have been described as very promising antimicrobial agents [3, 4, 5]. Because of their antibacterial, antiviral, and antifungal properties as well as low background toxicity toward host cells, PA and their derivatives have found widespread applications as preservatives in food, pharmaceutical, and cosmetic products [6]. In addition to these properties, PA have been also described for other health protective effects such as antioxidants and anti‐inflammatory, therefore constituting a promising class of compounds for therapeutic applications [7].

In the context of TB, several PA have been identified with good anti‐TB properties [5, 8, 9, 10, 11] and thus proposed as potential candidates in TB therapy [5]. Historically, salicylic acid (SA) and its analogue, the 4‐amino salicylic acid also known as *para*‐amino salicylic acid (PAS), have been identified as potential antitubercular in the 1940s [12, 13, 14, 15]. As a direct consequence, PAS was then introduced in anti‐TB therapies and has been proven to be effective to cure TB when administered alone or coadministered with streptomycin to prevent resistance emergence [9, 13]. This was before the development of the current standard anti‐TB regimen; nevertheless, PAS remains in the anti‐TB arsenal and continues to be used nowadays as second‐/third‐line drug in some cases [16].

Recently, Zhang *et al*. [9] reported that *M. tuberculosis* was highly susceptible to a wide range of weak acids, including several PA compounds such as benzoic acid (BA), SA, or the anti‐inflammatory drug acetyl‐salicylic acid known as aspirin (ASP). Complementary studies focusing on other pathogenic species have also reported that SA and ASP could be used alone or in combination with known antibiotics to enhance antimicrobial susceptibility using *in vitro* and *in vivo* biological systems [17, 18, 19]. However, whether such strategy could be applied to *M. tuberculosis* with established molecules and/or new analogs has not been thoroughly investigated.

In that context, we sought to test the antibacterial activity of a panel of PA, including SA derivatives, against four pathogenic mycobacterial model species, including *M. tuberculosis*. Using this approach, we demonstrated that parental SA, as well as some iodinated derivatives, display promising antitubercular activities. Based on their chemical structures and their well‐established weak acid properties, we have tested the pH‐dependency of SA and its derivatives and further established that some of them exhibit significant pH‐driven activities. By using a genetically encoded mycobacterial intrabacterial pH reporter, we describe for the first time that SA, iodinated SA‐derivatives, and the anti‐inflammatory drug ASP are able to disrupt the intrabacterial pH of *M. tuberculosis* in a dose‐dependent manner under *in vitro* conditions mimicking the endolysosomal pH of macrophages. Conversely, the structurally related second‐line anti‐TB drug PAS revealed different inhibitory profiles, a strong antagonism with l‐methionine supplementation and no pH‐dependency suggesting distinct modes of action. Finally, we propose a model that could explain how SA and some noncytotoxic SA derivatives restrict *M. tuberculosis* growth in a pH‐dependent manner by acidifying its cytosol, therefore making such compounds very attractive for further development.

## Methods

### Chemical compounds and computed properties

Compounds gallic acid (GA), iodo‐gallic acid (IGA), *trans*‐ferulic acid (FA), and 2‐iodo‐*trans*‐ferulic acid (2IFA) were obtained as described previously [20]. Salicylic acid (SA; #247588), 5‐iodo‐salicylic acid (5ISA; #I10600), 3,5‐diiodo‐salicylic acid (3,5diISA; D124001), 5‐iodo‐2‐methoxy benzoic acid (2M,5IBA; #754862), 4‐aminosalicylic acid (PAS; #A79602), and acetyl‐salicylic acid (ASP; #PHR1003) were purchased from Sigma‐Aldrich (Saint‐Quentin Fallavier, France) and were > 95% purity. Stock solutions in which the compounds were found to be completely soluble in DMSO (DASIT group; #455103) were prepared at a 20 mg·mL^−1^ final concentration (except for ASP which was concentrated at 10 mg·mL^−1^), prior to drug susceptibility testing.

Computed properties of all phenolic derivatives, such as molecular weight, octanol–water partition coefficient Log*P*, phenolic, and acidic p*K*_a_, were done using the Marvin Desktop Suite, Calculator Plugins 24.1.3 developed by ChemAxon (http://www.chemaxon.com).

### Bacterial strains and culture conditions

*Pseudomonas aeruginosa* PAO1 and *Escherichia coli* ATCC 25922 were routinely grown in Mueller–Hinton II broth (MHIIB; #90922; Sigma‐Aldrich) at 37 °C under agitation at 180 r.p.m.

*Mycobacterium abscessus* CIP104536^T^ smooth (S) or rough (R) morphotype, *Mycobacterium marinum* ATCC BAA‐535/M, and *M. tuberculosis* ATCC 25177 H37Ra reference strains were routinely grown in Middlebrook 7H9 broth (#271310; BD Difco, Le Pont de Claix, France) supplemented with 0.2% glycerol (#EU3550; Euromedex, Souffelweyersheim, France), 0.05% Tween‐80 (#P1754; Sigma‐Aldrich), and 10% oleic acid, albumin, dextrose, catalase (OADC enrichment; #211886; BD Difco). *Mycobacterium smegmatis* mc^2^155 reference strain was cultured in the same medium devoid of OADC supplementation. All cultures were maintained at 37 °C with shaking, except for *M. marinum*, which was cultured at 32 °C.

Recombinant *M. tuberculosis* H37Ra strain expressing pH‐GFP [21] (pUV15‐pHGFP; Addgene (Watertown, MA, USA) plasmid #70045, kindly gifted by S. Ehrt) was generated by electroporation according to Goude *et al*. [22] and further selected onto 7H11 agar medium (#283810; BD Difco) supplemented with 10% OADC and 50 μg·mL^−1^ of hygromycin B (#H007; Toku‐E, Sint‐Denijs‐Westrem, Belgium). Hygromycin B was used as selection marker for the culture maintenance of the fluorescent strain at a final concentration of 50 μg·mL^−1^ but was not used when performing intrabacterial pH homeostasis disruption experiments.

### Antimicrobial susceptibility testing on Gram^(−)^ bacteria

Minimum inhibitory concentrations were determined using the rapid INT colorimetric assay [23]. Briefly, fresh mid‐log phase bacterial culture (OD_600_ = 0.6–0.8) was diluted to a cell density of 1 × 10^6^ CFU·mL^−1^ in MHIIB. Then, 100 μL of this inoculum was added in a 96‐well flat‐bottom Corning^®^ microplates with lid (#CLS3370; Merck KGaA, Darmstadt, Germany) containing twofold serial dilutions of each compound to a final volume of 200 μL. Growth controls containing no compound (i.e., bacteria only = B), inhibition controls containing standard drug (= doxycycline), and sterility controls (i.e., medium only) without bacteria were also included. Plates were incubated at 37 °C for 16–18 h, then 50 μL of a *p*‐iodonitrophenyltetrazolium violet (INT) (#I8377; Sigma‐Aldrich) solution (0.2 mg·mL^−1^) was added to each well. The microplates were re‐incubated in the dark at 37 °C for 30 min until the appearance of a color change in the control B‐wells (bacteria alone). In the presence of active dehydrogenases, colorless INT solution is reduced to an insoluble purple formazan dye, synonymous of the presence of metabolically active bacteria. The absorbance of formazan was further measured at 470 nm with a Tecan Infinite^®^ 200 PRO multimode microplate reader (Tecan Group Ltd., Männedorf, Switzerland). Relative absorbance units were defined as: RAU% = (test well *A*_470 nm_/mean *A*_470 nm_ of control B wells) × 100. MIC values were determined by fitting the RAU% sigmoidal dose–response curves in the kaleidagraph 4.2 software (Synergy Software, Reading, PA, USA). The drug concentration that caused a 90% reduction in optical density compared to the growth controls was defined as the MIC_90_. All experiments were performed independently at least three times.

### Antimycobacterial susceptibility testing using resazurin microtiter assay (REMA)

Antimycobacterial susceptibility testing was performed using the Middlebrook 7H9 broth microdilution method. MICs were determined in 96‐well flat‐bottom Nunclon Delta Surface microplates with lid (#167008; Thermo‐Fisher Scientific, Illkirch, France) using the resazurin microtiter assay (REMA) [24, 25]. Briefly, log‐phase bacteria were diluted to a cell density of 5 × 10^6^ CFU·mL^−1^ in complete 7H9 medium. Then, 100 μL of the above inoculum was added to each well containing 100 μL of complete 7H9 medium, serial twofold dilutions of the compounds or controls, to a final volume of 200 μL (final bacterial load of 5 × 10^5^ CFU per well). Growth controls containing no inhibitor or with the DMSO vehicle (i.e., bacteria only), inhibition controls containing 50 μg·mL^−1^ kanamycin (#UK0010D; Euromedex), and sterility controls (i.e., medium only) without inoculation were also included. Microplates were incubated at 37 °C (32 °C for *M. marinum*) in a humidity chamber to prevent evaporation for 3–5 days (*M. smegmatis* and *M. abscessus*) or 10–14 days (*M. marinum* and *M. tuberculosis*). Then, 20 μL of a 0.025% (*w/v*) resazurin solution in sterile water (#R7017; Sigma‐Aldrich, Saint‐Quentin Fallavier, France) was added to each well, and the plates were incubated at 37 °C (or 32 °C) until color change from blue to pink or violet in the control well (i.e., bacteria alone). Fluorescence units (FU) of the metabolite resorufin (λ_ex_/λ_em_ = 530/590 nm) were quantified using a Tecan Spark 10M™ multimode microplate reader (Tecan Group Ltd). Relative fluorescence units (RFU) were defined as: RFU% = (test well FU/mean FU of control B wells) × 100. MIC values were determined by fitting the RFU% sigmoidal dose–response curves in the kaleidagraph 4.2 software. The lowest compound concentration leading to 90% inhibition of bacterial growth was defined as the MIC_90_. All experiments were performed independently at least three times.

### Cell culture and cytotoxicity assays

The cytotoxicity of compounds against eukaryotic cells was measured based on the reduction in resazurin [26, 27] as a value of cellular viability by metabolic activity. Murine macrophage cell‐line Raw264.7 (TIB‐71; American Type Culture Collection [ATCC], Manassas, VA, USA) were cultured from a cryo‐preserved stock in Dulbecco's modified Eagle medium (DMEM; #10‐013‐CV; Corning) supplemented with 10% heat‐inactivated fetal calf serum (FBS, #F7524; Sigma) in 25‐cm^2^ tissue culture flasks (#353108; Corning‐Falcon, Corning, NY, USA). Cells were grown at 37 °C and 5% CO_2_ until reaching subconfluency (60–80%).

For cytotoxicity experiments, approximately 1 × 10^5^ cells were seeded in each well of a 96‐well flat‐bottom Nunclon Delta Surface microplates (#167008; Thermo‐Fisher Scientific) in a final volume of 200 μL, and incubated for additional 16–24 h. Then, the medium was removed by aspiration, and 200 μL of serial twofold dilution of each compound in DMEM‐FBS were added to each well. After 24 h of incubation, 20 μL of a 0.025% (*w/v*) resazurin solution was added to each well. Fluorescence was measured following incubation for ~ 4 h at 37 °C and 5% CO_2_ in the dark, as described above, leading to relative metabolic activities. DMSO‐treated cells were used as 100% viability control conditions, and addition of 0.2% Triton X‐100 solution served as positive control of total lysis (0% viability). The compound concentration leading to 50% macrophages cell death was defined as the CC_50_. All experiments were performed in technical triplicates on two independent assays.

### pH‐dependent growth inhibition assay and MIC determination

Susceptibility testing was performed as described above with slight modifications. All experiments at pH 6.8 were performed in standard Middlebrook 7H9 containing 0.2% glycerol and 10% OADC enrichment. To avoid Tween‐80‐mediated toxicity, 0.025% Tyloxapol (#T8761; Sigma‐Aldrich) was used. For pH‐dependent experiments, the same media containing additional 50 mm of MES and adjusted at pH 5.5 [28, 29] was used. The drug of interest was twofold serial diluted in the appropriate media to a final volume of 100 μL per well in technical triplicate per microplate. Then, each well was inoculated with 100 μL (5 × 10^6^ CFU·mL^−1^) of a *M. tuberculosis* culture in pH 6.8 or pH 5.5‐ajusted Middlebrook 7H9. Positive growth controls (i.e., inoculum without antibiotics or with DMSO), positive growth inhibition controls (i.e., 50 μg·mL^−1^ kanamycin), and sterility controls (i.e., medium only) were included. The 96‐well flat‐bottom Nunclon Delta Surface microplates were incubated at 37 °C during 14–21 days for both pH 6.8 and pH 5.5 experiments. MIC of each antibiotic at each pH was determined by visual inspection according to EUCAST recommendations [30], and by absorbance reading at 600 nm to confirm visual recording. The first antibiotic concentration that visually inhibited bacterial growth was defined as the MIC_90_.

### Intrabacterial pH homeostasis disruption assays

Recombinant *M. tuberculosis* harboring pUV15‐pHGFP and producing the ratiometric pH‐sensitive pH‐GFP sensor was used for the determination of intrabacterial pH homeostasis disruption [21, 31, 32]. Briefly, exponentially growing *M. tuberculosis* cultures were centrifuged at 2700 *g* during 5 min, and resuspended in 10 mL of Middlebrook 7H9 adjusted at pH 5.5 or pH 6.8 in order to have a normalized OD_600 nm_ of 0.8. Then, 100 μL of this bacterial inoculum was used to inoculated wells containing 100 μL of serial‐diluted compounds of interest, giving a final OD_600 nm_ of 0.4 in each well. Prior performing the intrabacterial pH (IBpH) perturbation experiments, we have checked that all the compounds, at their highest concentration tested, did not impact the pH of the medium by more than 0.1 pH unit. The pH‐GFP fluorescence was collected at λ_em_ 535 nm after excitation at λ_ex_ 405 nm and λ_ex_ 488 nm, respectively, using a TECAN Spark 10M™ multimode microplate reader (Tecan Group Ltd). For all our experiments at pH 5.5, the well‐established ionophore Monensin (#M5273; Sigma) was used at 20 μm as internal positive control of intrabacterial pH homeostasis disruption. For all our experiments at pH 6.8, the well‐established protonophore CCCP (#215911; Merck) was used at 100 μm as internal positive control of intrabacterial pH homeostasis disruption. The ratios in fluorescence intensity λ_ex_ 405 nm/λ_ex_ 488 nm from each condition were calculated. All the results were exported as CSV files, imported in the r studio software (The R Project for Statistical Computing, version 1.3.1073), and graphs were plotted with the ggplot2 package (version 3.3.2). Determination of half‐maximal effective concentration (EC_50_) was performed in the r studio software using a four‐parameter logistic nonlinear regression model [32]. Finally, a calibration curve of the pH‐GFP sensor was performed as previously described [21, 31, 33] by lysing *M. tuberculosis* pH‐GFP cells in 1 mL of citrate–phosphate buffers adjusted with pH ranging from 8 to 5.5 by increments of 0.5. Clarified lysates were assessed for fluorescence intensities at an emission wavelength λ_em_ 535 nm after excitation at λ_ex_ 410 nm and λ_ex_ 480 nm. Recording was performed using a TECAN Spark 10M™ multimode microplate reader (Tecan Group Ltd), and a fluorescence ratio λ_ex_ 410 nm/λ_ex_ 480 nm was calculated. These data allowed to build a calibration curve, using the LOESS (locally estimated scatterplot smoothing) prediction model in R to fit a smooth curve on our data and provide an accurate estimation of *M. tuberculosis* IBpH.

### Spontaneous resistant mutant isolation

Approximately, 10^6^ CFU of exponentially growing *M. tuberculosis* H37Ra were plated on a single Petri dish of Middlebrook 7H11 agar medium supplemented with 10% OADC and increasing concentrations of SA, 5ISA, 3,5diISA, ASP, or PAS, respectively (corresponding to 1×, 2× or 5× MIC_90_ at pH 6.8). Additional plates without compounds were included in the experiments as growth control. Plates were incubated at 37 °C and a thorough visual inspection of resistant clones' appearance was done after 4 and 6 weeks. Experiments were performed at least on two independent biological replicates.

### Quantification and statistical analysis

All results displayed in this study were obtained from *n* = 2 or *n* = 3 biologically independent experiments performed at least each time in three technical replicates (unless otherwise stated). For statistical analysis in the intrabacterial pH homeostasis disruption assays, the means between the conditions of interest were tested for significant differences using one‐way ANOVA followed by Tukey's posttest with the “aov()” and “TukeyHSD()” functions in R – r studio using the ggpubr R package. All the *P*‐values contained in the text or the figures are relative to the control condition (unless otherwise stated). All *P*‐values were considered significant when *P*‐value < 0.05. Statistical analysis is displayed in the figures as: n.s, not significant; *P*‐value < 0.05; *P*‐value < 0.01; or *P*‐value < 0.001. In each figure or table legend, the statistical tests used, the number of biologically independent replicates and the number of technical replicates is indicated.

## Results and Discussion

### Screening and identification of inhibitory properties of a small subset of PA on mycobacterial model species

We recently described the antibacterial activity of a subset of PA and iodinated‐PA on the Gram^(+)^ opportunistic pathogen *Staphylococcus aureus* [20]. Results showed that although native PA did not impact growth at concentration up to 1 mm (i.e., 140–200 μg·mL^−1^), some of their iodinated derivatives displayed better inhibitory activities with minimal inhibitory concentration (MIC) values comprised around 400–700 μm (i.e., 120–180 μg·mL^−1^) [20]. Based on these observations, we have conducted a more comprehensive susceptibility testing investigation of both parental and iodinated forms of PA against the Gram^(−)^ bacteria *E. coli* and *P. aeruginosa*; and four mycobacterial species, which include the saprophytic species *M. smegmatis*, the opportunistic pathogens *M. marinum* and *M. abscessus* and finally the tubercle bacilli, *M. tuberculosis*.

**Fig. 1:**
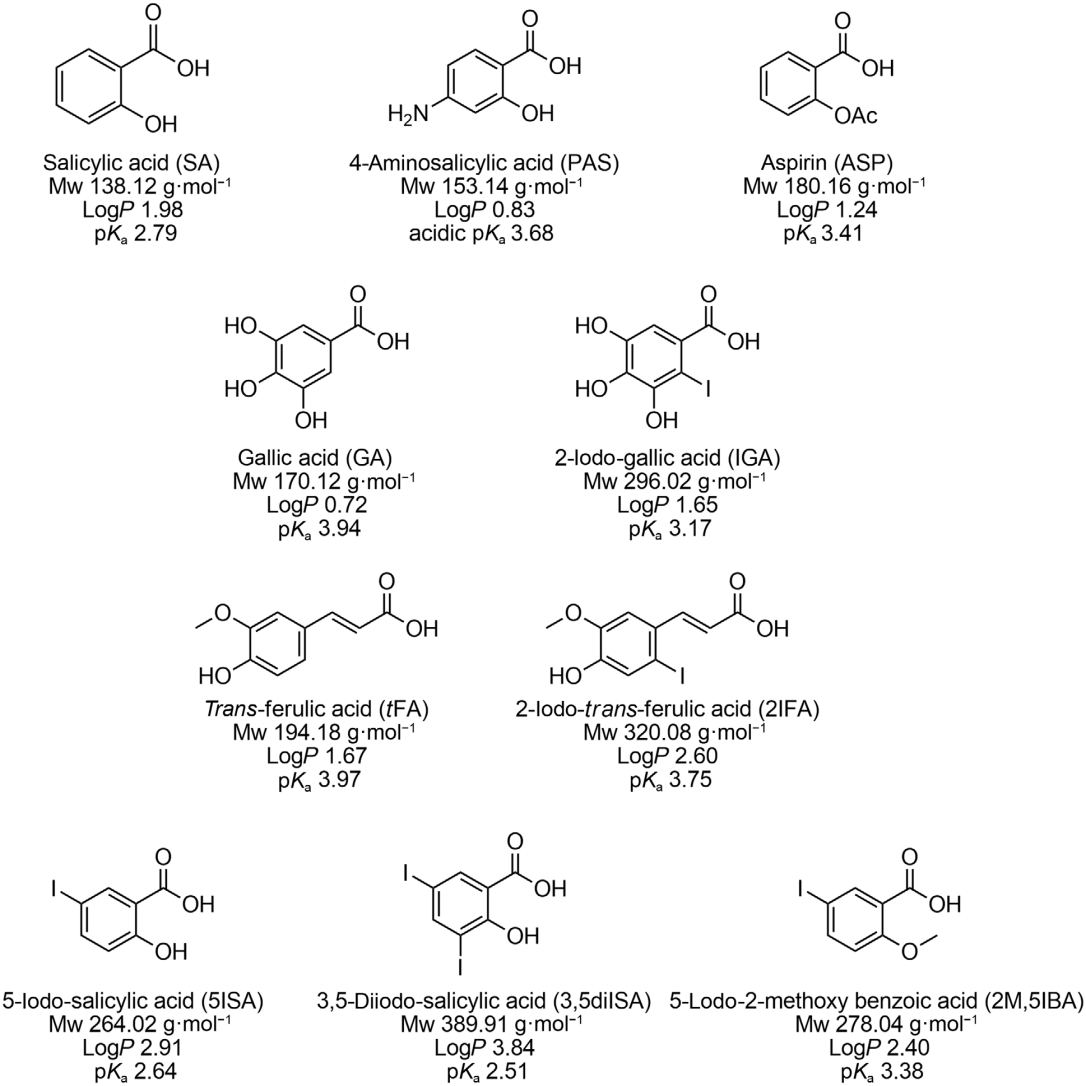
Structure and physico‐chemical properties of the PA compounds investigated in this study.

The chemical structure of the eight compounds tested, gallic acid (GA), 2‐iodo‐gallic acid (IGA), *trans*‐ferulic acid (*t*FA), 2‐iodo‐ferulic acid (2IFA), salicylic acid (SA), 5‐iodo‐salicylic acid (5ISA), 3,5‐diiodo‐salicylic acid (3,5diISA), and 5‐iodo‐2‐methoxy benzoic acid (2M,5IBA) are displayed in Fig. 1, and their inhibitory parameters obtained by performing susceptibility testing are reported in Table 1.

**Table 1:** Antibacterial activities of the eight phenolic compounds compared to standard antimicrobial agents against two Gram^(−)^ bacteria and four mycobacterial strains. The best MIC values obtained against *M. tuberculosis* H37Ra are highlighted in bold. AMK, Amikacin = reference drugs; DOX, doxycycline; INH, isoniazid; NT, not tested (not enough compound).

Compounds	MIC_90_ (μg·mL^−1^)^a^							CC_50_ (μg·mL^−1^)^b^
	*P. aeruginosa* PAO1	*E. coli* ATCC 25922	*M. smegmatis* mc^2^155	*M. abscessus* CIP 104536^T^		*M. marinum* M	*M. tuberculosis* H37Ra	
				S variant	R variant			
Gallic acid (GA)	> 500	> 500	> 500	> 500	> 500	> 500	> 500	155
2‐Iodo‐gallic acid (IGA)	> 500	> 500	> 500	> 500	> 500	> 500	> 500	> 500
*Trans*‐ferulic acid (*t*FA)	> 500	> 500	> 500	> 500	> 500	> 500	> 500	> 500
2‐Iodo‐*trans*‐ferulic acid (2IFA)	> 500	> 500	> 500	NT	NT	NT	> 500	> 500
Salicylic acid (SA)	> 500	> 500	> 500	> 500	> 500	> 500	**104**	> 500
5‐Iodo‐salicylic acid (5ISA)	> 500	283	> 500	223	484	283	**58.5**	> 500
3,5‐Diiodo‐salicylic acid (3,5diISA)	> 500	> 500	221	314	> 500	382	**64.4**	314
5‐Iodo‐2‐methoxy benzoic acid (2M,5IBA)	> 500	> 500	> 500	> 500	> 500	> 500	486	> 500
DOX	4	16	–	–	–	–	–	–
INH	–	–	1.5	–	–	9.2	0.15	> 20^c^
AMK	–	–	–	3.4	5.9	1.7	0.37	> 90^c^

^a^ Experiments were performed as described in the Methods section. MIC_90_: minimum inhibitory concentration leading to 90% growth inhibition as determined either by the INT assay (Gram^(−)^ bacteria) or by the REMA assay (mycobacterial species), and are expressed as mean values of three independent assays performed in duplicate (CV% < 5%)

^b^ CC_50_: Cytotoxic concentrations leading to 50% Raw264.7 macrophages cell death as determined by the REMA assay; values are the mean of a two dose–response experiments carried out in triplicate

^c^ Data from [34].

First, susceptibility testing against the two Gram^(−)^ bacterial strains, *E. coli* and *P. aeruginosa*, showed that the eight molecules were poorly active against the two strains with MIC_90_ values ranging from 283 μg·mL^−1^ to higher than 500 μg·mL^−1^ (Table 1). Further investigation and analysis of fitted growth inhibition profiles and MIC_90_ values against mycobacteria demonstrated that GA, IGA, *t*FA and 2IFA compounds were almost completely inactive against the species tested with MIC_90_ values superior to 500 μg·mL^−1^ (Table 1). On the other hand, SA and its derivatives displayed heterogeneous growth inhibitory activities across the various species with MIC_90_ ranging from 58.5 and up to 486 μg·mL^−1^ (Table 1). Of importance, *M. tuberculosis* was found to be more susceptible to iodinated SA‐derivatives than *M. smegmatis*, *M. abscessus* S and R morphotypes and *M. marinum*. In fact, 5ISA and 3,5diISA strongly inhibited the growth of *M. tuberculosis* with relatively good MIC_90_ values of 58.5 and 64.4 μg·mL^−1^, respectively, which are 3.4–8.3 times lower than those obtained for the other mycobacterial strains. As previously observed with *S. aureus* [20], insertion of iodine atoms slightly increased the antibacterial activity of the resulting iodinated derivatives *versus* the parental SA molecule (MIC_90_ = 104 μg·mL^−1^) against *M. tuberculosis*.

Finally, the toxicity of the eight PA against murine Raw264.7 macrophages was investigated using a classical dose–response assay [26]. The calculated response parameter was the CC_50_, which corresponds to the concentration required to elicit a 50% decrease in cell viability compared with the control (Table 1). Except for GA (CC_50_ = 155 ± 16 μg·mL^−1^) and 3,5diISA (CC_50_ = 314 ± 18 μg·mL^−1^) for which very moderate levels of toxicity were found, the other compounds were not toxic to Raw264.7 cells, as demonstrated by CC_50_ values > 500 μg·mL^−1^. This finding is in line with previous results showing that these compounds do not affect the viability of L929, HeLa, T84, and Caco‐2 cells [20].

Given all these findings, amongst the eight tested phenolic compounds, SA and its two iodinated derivatives, 5ISA and 3,5diISA, were displaying the best combination of antibacterial activities against *M. tuberculosis* and cytotoxic properties, and were then selected for further mechanistic investigations.

### Analysis of pH‐driven activities of SA and SA derivatives

Previous report demonstrated that SA and ASP antibacterial activities against *M. tuberculosis* were moderate. However, similar to many weak acids, both compounds displayed interesting pH‐driven potency against *M. tuberculosis* [9]. Indeed, according to their chemical properties and the Henderson–Hasselbalch equation, a decrease in extrabacterial pH should increase the proportion of neutral protonated weak acid [AH], whereas an increase in pH would favor the formation of its negatively charged anion [A^−^], as described for the anti‐TB drug pyrazinoic acid [9, 35]. Thus, at acidic pH, the increase in the protonated neutral form might have two major effects that could explain a more potent activity. First, protonation of weak acids to their neutral form has been shown to facilitate their ability to cross biological membranes [36, 37, 38], thereby increasing their intrabacterial concentrations. Second, according to this weak acid model, protonated forms may have the ability to unilaterally translocate protons through the mycobacterial envelope by passive diffusion in a pH‐dependent manner, and, based on the p*K*_a_ of the carboxylic acid function, subsequently release protons in the pH‐neutral bacterial cytosol [9, 35, 36].

In that context, we explored whether SA, its two iodinated derivatives, 5ISA and 3,5diISA as well as the FDA‐approved references drugs PAS and ASP might share a similar primary mode of action and commonly display pH‐driven activities. To achieve this goal, we first assessed antibacterial activity in standard 7H9 medium at pH 6.8 and in MES‐adjusted pH 5.5 medium [28]. Since Tween‐80 has been shown to impact viability at low pH, this detergent was replaced in both media by tyloxapol, a nonhydrolysable nontoxic dispersible agent [21]. The results obtained in 7H9 medium at nearly neutral pH 6.8 in this slightly different methodological system confirmed those obtained above during the multispecies screening, with the SA derivatives showing MIC_90_ values in the range of 100–200 μg·mL^−1^ (Table 2).

**Table 2:** Antibacterial activities of SA derivatives on *Mycobacterium tuberculosis* at near‐neutral (pH 6.8) and acidic (pH 5.5) pH in the presence or absence of the PAS antagonist l‐methionine.

Compounds	*M. tuberculosis* H37Ra – MIC_90_ (μg·mL^−1^)^a^				
	pH 6.8	pH 5.5	Fold change pH 6.8/pH 5.5	pH 6.8 + l‐methionine	pH 5.5 + l‐methionine
Salicylic acid (SA)	200	25	×8	200	6.25
5‐Iodo‐salicylic acid (5ISA)	100	50	×2	100	25
3,5‐Diiodo‐salicylic acid (3,5diISA)	200	100	×2	200	50
Acetyl‐salicylic acid (ASP)	250	31.25	×8	250	31.25
4‐Amino‐salicylic acid (PAS)	0.31	0.31–0.63	×1–/2	> 20	> 20

^a^ Experiments were performed as described in the Methods section. MIC_90_: Minimum inhibitory concentration leading to 90% growth inhibition as determined by visual inspection and confirmed by absorbance reading at 600 nm, are expressed as mean values of at least three independent dose–response experiments carried out in technical triplicate.

In contrast, performing the experiments at acidic pH 5.5 revealed a strong compound‐dependent acidic pH‐mediated potentiation, with changes in MIC_90_ from two‐ to eightfold (Table 2). The best effect was observed with the reference compound SA with an eightfold decrease in MIC_90_, shifting from 200 to 25 μg·mL^−1^. This latter result is in perfect agreement with previously published observations from Zhang *et al*. [9] who reported a better anti‐TB activity of SA at acidic pH 5.5 (MIC_90_ = 10–20 μg·mL^−1^) than at neutral pH 6.8 (MIC_90_ of 50–100 μg·mL^−1^). Determination of the MIC_90_ of ASP showed a very similar pattern, with MIC_90_ values at pH 6.8 at 250 and 31.25 μg·mL^−1^ when assessed at pH 5.5 resulting in a an eightfold decrease.

Regarding the two iodinated derivatives, 5ISA and 3,5diISA, a twofold decrease in MIC_90_ values was observed at pH 5.5 *vs*. pH 6.8 (Table 2). Analysis of the calculated p*K*_a_ of each compound (Fig. 1) failed to establish a clear quantitative link between the observed pH dependence and the ability of the COOH function to gain protons [9], suggesting that more complex or multiple parameters may influence the activities of these compounds.

As expected, the FDA‐approved anti‐TB drug PAS displayed the best activity with MIC_90_ value of 0.31 μg·mL^−1^ when assessed at pH 6.8 and was not significantly impacted by acidic pH, suggesting that PAS does not act as a pH‐dependent compound (Table 2).

Altogether, our results confirm that SA and ASP exhibit strong pH‐dependent potentiation [9], whereas iodinated analogs are only slightly affected by this parameter. This was also observed for PAS which consistently inhibited bacterial growth regardless of environmental pH. Such findings confirm that structural analogs, with very minor differences can still exhibit different pH‐dependent inhibitory activity.

### Methionine‐mediated antagonism occurs for the folate biosynthesis targeting compound PAS but not any other SA derivatives

Recent studies have demonstrated that l‐methionine supplementation can drastically affect the antibacterial activity of PAS and other antifolate drugs [39, 40]. To gain further insight into the mode of action of SA and its derivatives, we tested whether l‐methionine could also have a negative impact on their inhibitory features at both near‐neutral and acidic pH. Of note, although l‐methionine supplementation has been widely described and used as potent antagonist of PAS by bypassing the folate pathway, we cannot rule out that this molecule may have alternative effects on mycobacterial physiology in our assay.

As expected, supplementation with 10 μg·mL^−1^ of l‐methionine triggered an important change in PAS inhibitory activity with MIC_90_ shifting from 0.3125 to > 20 μg·mL^−1^ at both pH 6.8 or pH 5.5 (Table 2). This 64‐fold increase in MIC_90_ is in perfect agreement with previously published observations from Howe *et al*. [40]. In contrast, in the presence of l‐methionine at both pH, identical or a twofold decrease in MIC_90_ values were observed for ASP, 5ISA, and 3,5diISA (Table 2). Surprisingly, l‐methionine supplementation potentiated the activity of SA by fourfold at acidic pH but not at near‐neutral pH (Table 2). This result was not really expected, but the beneficial effect of l‐methionine has been already reported in the literature [41]. Indeed, l‐methionine has been shown to potentiate several classes of antibiotics including macrolides, cyclines, or fluoroquinolones, and such process has been proposed to be associated with a decrease in efflux and/or alteration of the oxidative stress [41].

Taken together, our results suggest that the mode of action of SA, 5ISA, 3,5diISA, and ASP may be clearly distinct from that of PAS which is strongly antagonized by l‐methionine. Accordingly, we propose that the *M. tuberculosis* folate pathway is unlikely to be targeted by SA, 5ISA, 3,5diISA, and ASP.

### SA and SA derivatives acidify *M. tuberculosis* cytosol in a dose‐dependent and pH‐dependent manner

**Fig. 2:**
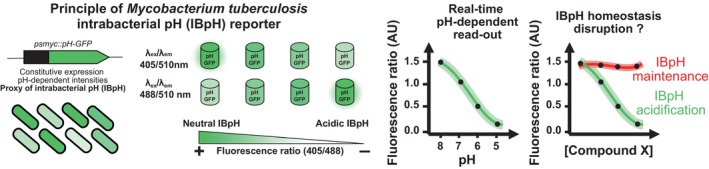
Schematic representation of *Mycobacterium tuberculosis* intrabacterial pH (IBpH) homeostasis monitoring in real time using the genetically encoded pH‐GFP sensitive ratiometric reporter. The *pH‐GFP* gene is encoded by the pUV15‐pHGFP episomal vector and its expression is driven under the strong constitutive promoter *psmyc*. The dual pH‐GFP excitation/emission wavelengths are inversely responding to pH exposure, thereby displaying distinct fluorescence intensity profiles which can be monitored noninvasively overtime. Analysis can be expressed as fluorescence intensity ratio that are obtained by dividing the fluorescence intensity acquired with excitation/emission channels of 405/510 nm by the one at 488/510 nm. A decrease in the 405/488 nm ratio highlights the acidification of the bacterial cytosol. A representation of such decrease in ratiometric signal quantification as function of pH is displayed. Finally, this tool can be used to monitor the effect of drugs onto *M. tuberculosis* IBpH homeostasis as highlighted on the far‐right panel.

We capitalized from a well‐established genetically encoded intrabacterial pH (IBpH) reporter (Fig. 2) [21, 31, 32] to assess whether SA and SA‐iodinated derivatives would be able to acidify IBpH as previously hypothesized in the case of weak acids [9].

**Fig. 3:**
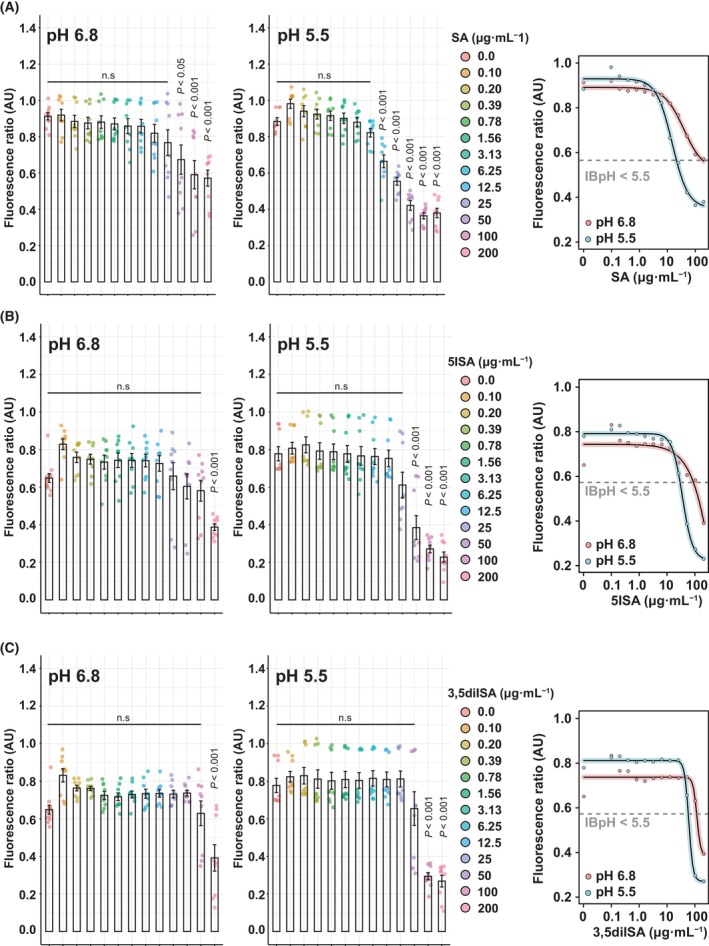
*Mycobacterium tuberculosis* intrabacterial pH homeostasis disruption by SA and derivatives. (A–C) Quantification of *M. tuberculosis* pH‐GFP ratio (405/488 nm) in the presence of increasing concentrations of (A) SA, (B) 5ISA and (C) 3,5diISA for 24 h. Ratiometric signals were obtained by dividing the fluorescence intensity acquired with excitation/emission channels of 405/510 nm by the one obtained at 488/510 nm. Results from SA, 5ISA and 3,5diISA are displayed from top to bottom, respectively. Analysis performed at pH 6.8 are displayed on the left panels whereas analysis performed at pH 5.5 are displayed on the middle panels. The right panels correspond to the 4‐parameter nonlinear logistic regression of the data displayed in the left and middle panels respectively. Results were obtained from *n* = 3 biologically independent experiments and are displayed as mean ± SEM. Statistical significance was assessed by comparing the means of each concentration with its respective control condition using one‐way ANOVA followed with Tukey's multiple comparisons test. All *P*‐values were considered significant when *P*‐value < 0.05. EC_50_ determination was not applicable for the compound 5ISA at pH 6.8 due to a lack of complete sigmoidal response. IBpH values are expressed as pH units and were calculated using a calibration curve of the sensor. The gray dotted lines show the IBpH threshold of 5.5.

Dose–response analysis performed at pH 6.8 showed that only very high concentration (50–200 μg·mL^−1^) of SA triggers IBpH homeostasis perturbation (Fig. 3A—left panel). Indeed, such process was only visible at concentrations close to SA's MIC_90_ value with statistically significant changes noticeable when pulsed with concentrations comprised between 50 and 200 μg·mL^−1^ (*P*‐value < 0.05 and *P*‐value < 0.001, respectively). Increasing protons availability in the extracellular medium by adjusting the pH at 5.5 significantly increased the ability of SA to disrupt IBpH homeostasis. Indeed, concentrations as low as 12.5 μg·mL^−1^ significantly affected the fluorescence ratio, thus supporting acidification of the bacterial cytosol (Fig. 3A—middle panel). Dose response curve analysis and 4‐parameter regression allow us to estimate SA's EC_50_ for IBpH homeostasis disruption. As shown in Fig. 3A (right panel), acidic pH potentiated the effect of SA on IBpH, with a drop in EC_50_ of approximately 2.5‐fold (38.9 ± 26.4 μg·mL^−1^ *vs*. 15.1 ± 1.7 μg·mL^−1^ at pH 6.8 and 5.5, respectively). This confirms the seminal observation of Zhang *et al*. [9] who reported that SA possesses a pH‐driven, pH‐disruptive effect against the tubercle bacilli.

The same approach was conducted with the 5ISA and 3,5diISA compounds (Fig. 3B,C). At pH 6.8, a significant perturbation of IBpH homeostasis was only visible at the highest concentration tested (200 μg·mL^−1^—*P*‐value < 0.001) (Fig. 3B,C—left panels). At pH 5.5, both derivatives disrupt IBpH homeostasis similarly to SA. This shift in *M. tuberculosis* iBpH was reached at lower concentrations of 50 and 100 μg·mL^−1^ for 5ISA and 3,5diISA, respectively (*P*‐value < 0.001) (Fig. 3B,C—middle panels). Similar trend was observed on their corresponding fitted graphs, highlighting a pH‐disruptive effect (Fig. 3B,C—right panels).

Overall, all these results confirm that the introduction of an iodine atom on the parent SA molecule had only a minor effect on its antibacterial activity. On the other hand, these iodinated derivatives retain their ability to be potentiated at acidic pH, and exhibit IBpH disruptive activities closely correlated with their ability to block bacterial growth.

### ASP, but not PAS, displays IBpH disruptive activity reflecting different modes of action

**Fig. 4:**
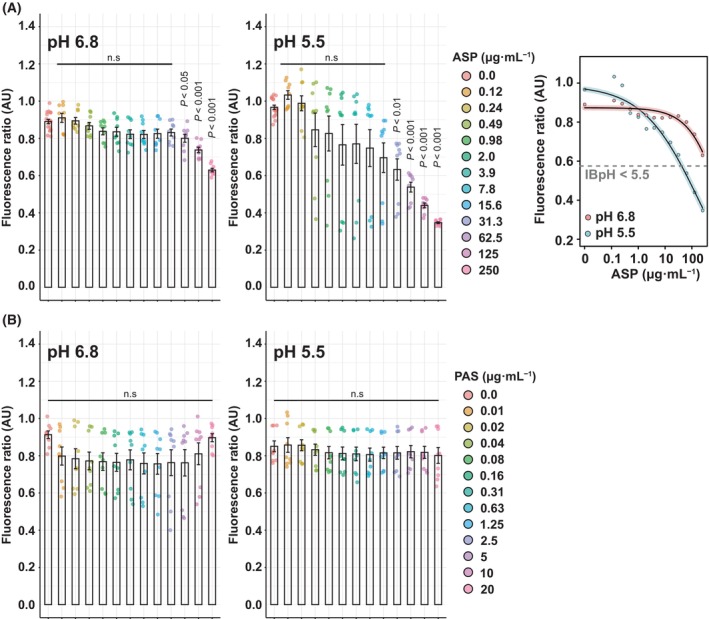
Divergent effects of the clinically available drug ASP and PAS on *Mycobacterium tuberculosis* intrabacterial pH homeostasis supports different antibacterial mode of action. (A, B) Quantification of *M. tuberculosis* pH‐GFP ratio (405/488 nm) in the presence of increasing concentrations of ASP (A) or PAS (B) for 24 h. Ratiometric signals were obtained by dividing the fluorescence intensity acquired with excitation/emission channels of 405/510 nm by the one obtained at 488/510 nm. Results from ASP and PAS are displayed from top to bottom respectively. Analysis performed at pH 6.8 are displayed on the left panels whereas analysis performed at pH 5.5 are displayed on the middle panels. Results were obtained from *n* = 3 biologically independent experiments and are displayed as mean ± SEM. Statistical significance was assessed by comparing the means of each concentration with its respective control condition using one‐way ANOVA followed with Tukey's multiple comparisons test. All *P*‐values were considered significant when *P*‐value < 0.05. The right panel of ASP corresponds to 4‐parameter nonlinear logistic regression of the data displayed in the left and middle panels respectively. No curve‐fitting was performed for PAS due to a lack of convergence when performing the 4‐parameter nonlinear logistic regression. IBpH values are expressed as pH units and were calculated using a calibration curve of the sensor. The gray dotted lines show the IBpH threshold of 5.5.

Finally, we tested the ability of the clinically relevant drugs, PAS and ASP, to disrupt *M. tuberculosis* IBpH (Fig. 4A,B). Structurally, ASP is a very close analogue of SA in which the hydrogen that is attached to the phenolic hydroxy group has been replaced by an acetyl group. With a strong pH‐dependent potency, we hypothesized that ASP may display an important IBpH disruptive activity similar to SA. The dose–response analysis performed showed that ASP had modest but significant effects on IBpH when tested at pH 6.8 and concentrations ranging from 62.5 to 250 μg·mL^−1^ (*P*‐value < 0.05 and *P*‐value < 0.001, respectively) (Fig. 4A). When the experiment was carried out at acidic pH, ASP was able to significantly disrupt IBpH from 31.25 μg·mL^−1^, supporting its previously observed pH‐dependent activity. Important changes were also observed at lower concentration such as 15.6 μg·mL^−1^ but failed to reach statistical significance with a *P*‐value of 0.06 (Fig. 4A).

On the contrary, PAS has no effect on IBpH at both pH 6.8 and pH 5.5, even when tested at 20 μg·mL^−1^, a concentration 64 times higher than its MIC_90_ (Fig. 4B). These results further indicate that the antitubercular activity of PAS does not definitively result from a major modification of the IBpH, but is likely due to the inhibition of folate biosynthesis by targeting the dihydrofolate reductase DfrA [42], as supported by our own observations of l‐methionine‐mediated antagonism (Table 2).

### Inability to isolate SA and SA‐derivatives spontaneous resistant mutants

Previous studies have reported that isolation of spontaneous resistant to weak acids is extremely complicated, likely due to their unique mode of action disrupting intrabacterial pH homeostasis independently of a specific protein target. In that context, Zhang *et al*. [9] suggested a potential highly conserved mechanism by which intracytoplasmic protons alter numerous processes simultaneously and therefore do not allow the selection of spontaneous resistant mutants.

To further characterize the mode of action of our compounds, we performed a similar subset of experiments and tried to isolate spontaneous resistant mutants as previously described [9, 43]. For that, 10^6^ CFU were plated on 7H11 agar plates at pH 6.8 containing a concentration corresponding to the MIC, 2 × MIC or 5 × MIC of compounds. After 4–6 weeks of incubation, no resistant colonies had appeared for the compounds that displayed pH‐driven, pH‐disruptive activities, suggesting that the isolation of resistant mutants is indeed complex and might require long‐term exposure at suboptimal concentration to select and enrich for mutations that increase fitness in the presence of weak acids as previously demonstrated [44].

Overall, these results do not necessary rule out that these compounds do not have specific target(s) in *M. tuberculosis*, but they are in favor of a potential pH‐dependent pH‐disruptive activity that precludes the selection of spontaneous resistant as previously suggested by Zhang *et al*. [9].

### SA and SA‐derivatives abilities to inhibit *M. tuberculosis* growth correlate with their IBpH homeostasis disruptive action

**Fig. 5:**
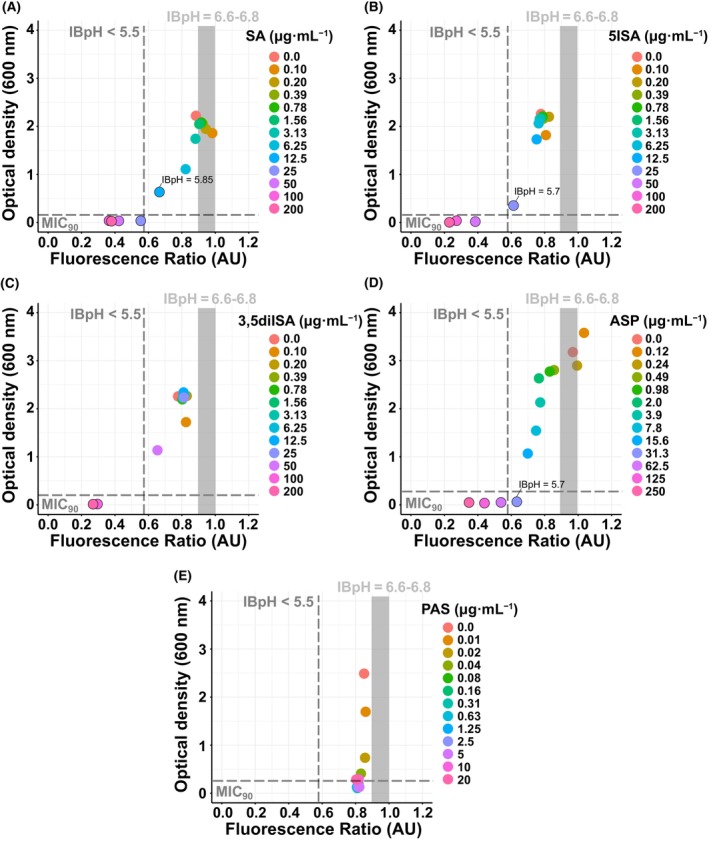
Antibacterial activities of SA and its derivatives correlate with *Mycobacterium tuberculosis* intrabacterial pH homeostasis perturbation. (A–E) Correlation analysis between *M. tuberculosis* pH‐GFP ratio (405/488 nm) at 24 h posttreatment (*x* axis) and *M. tuberculosis* growth after 21 days at pH5.5 (*y* axis) in the presence of increasing concentrations of (A) SA, (B) 5ISA, (C) 3,5diISA, (D) ASP and (E) PAS. The horizontal gray dotted line shows the MIC_90_ cut‐off values, representing 90% of growth inhibition compared to untreated control conditions. The vertical gray dotted lines show the IBpH value of 5.5, while the thick solid gray line represents the IBpH values of 6.6–6.8. Additional data may be shown on the graphs for values close to the MIC_90_ or IBpH < 5.5 cutoff.

Several independent groups, including ours, have reported that IBpH homeostasis inhibitors often display a strong correlation between their ability to alter intracytosolic pH and restrict bacterial growth [21, 31, 32, 33, 45, 46, 47]. To investigate whether there is a causal relationship between IBpH alteration and compound antibacterial activity, we compared the data obtained from *M. tuberculosis* antimicrobial susceptibility testing with those from IBpH homeostasis assays. For each compound of interest, the results presented in Fig. 5 show the growth of *M. tuberculosis* (OD_600 nm_) as function of its IBpH.

As expected, *M. tuberculosis* growth consistently decreased with increasing concentrations of compounds. Remarkably, for SA, 5ISA, 3,5diISA, and ASP this growth inhibition pattern was consistently associated with a sharp decrease in IBpH homeostasis. Analysis of the MIC_90_ values showed that, almost systematically, 90% of growth inhibition was achieved with concentrations that lowered the IBpH below 5.5. With SA, we indeed show that inhibition of *M. tuberculosis* growth from 25 μg·mL^−1^ correlates perfectly with a drop in IBpH below 5.5 (Fig. 5A). A clearly similar pattern was observed for 5ISA, 3,5diISA, and ASP, with concentrations that inhibited *M. tuberculosis* growth identical to those that disrupted its IBpH (Fig. 5B–D).

The inclusion of PAS in our assay confirmed that this compound is a very potent inhibitor of *M. tuberculosis* growth, even at concentrations below 1 μg·mL^−1^, but does not alter the IBpH. As shown in Fig. 5C, and as previously reported with other anti‐TB drugs [31, 32], the use of PAS clearly demonstrates that inhibition of *M. tuberculosis* growth does not systematically lead to IBpH alteration, whereas IBpH disruption always results in *M. tuberculosis* growth inhibition. Finally, this latter analysis also confirms that the mode of action of PAS is different from that of SA and iodinated derivatives, which act in a pH‐dependent manner.

## Conclusion

In this study, we have identified and characterized the pH‐dependent and IBpH‐disruptive activities of compounds that are derived from SA, a very simple PA. We demonstrate that iodination of SA does not alter the antibacterial activity of the resulting 5ISA and 3,5diISA derivatives, and could constitute an interesting way to synthetize new antibacterial molecules. In addition, our mechanistic studies reveal that the anti‐inflammatory drug ASP is also a potent anti‐TB drug that is potentialized at acidic pH and further acidify *M. tuberculosis* cytosol. This mode of action appears to be different from that of the well‐established anti‐TB drug PAS, which is also an analogue of SA. Altogether, these observations suggest that PA and more precisely SA‐derivatives may represent an interesting subset of molecules that could open new avenues for the development of new antibacterial entities with more potent activity in physiologically relevant microenvironments encountered by *M. tuberculosis*.

## Conflict of interest

The authors declare no conflict of interest.

## Author contributions

VS, PS, and J‐FC proposed, conceived, and led the study. VS and FM provided iodinated compounds. JL performed all pH‐dependent assays with the guidance of SC and PS. TF and EF performed the toxicity and MIC determination with the guidance of J‐FC, CC, and J‐MB. JL, PS, and J‐FC edited the figures. All authors provided intellectual input by organizing, analyzing, and/or discussing data. PS wrote the manuscript with input from JL and J‐FC. All authors have read the manuscript and provided critical feedback before its submission.

## Data Availability

Any additional data that support the findings of this study are available upon reasonable request from the corresponding authors at vadim.shlyonskiy@ulb.be, psantucci@imm.cnrs.fr, jfcavalier@imm.cnrs.fr.
